# Current Knowledge on Microviridin from Cyanobacteria

**DOI:** 10.3390/md19010017

**Published:** 2021-01-04

**Authors:** Samuel Cavalcante do Amaral, Patrick Romano Monteiro, Joaquim da Silva Pinto Neto, Gustavo Marques Serra, Evonnildo Costa Gonçalves, Luciana Pereira Xavier, Agenor Valadares Santos

**Affiliations:** 1 Laboratory of Biotechnology of Enzymes and Biotransformation, Biological Sciences Institute, Federal University of Pará, Belém 66075-110, Brazil; samuel.amaral@icb.ufpa.br (S.C.d.A.); patrick.monteiro@icb.ufpa.br (P.R.M.); joaquim.neto@icb.ufpa.br (J.d.S.P.N.); gustavo.serra@icb.ufpa.br (G.M.S.); lpxavier@ufpa.br (L.P.X.); 2 Laboratory of Biomolecular Technology, Biological Sciences Institute, Federal University of Pará, Belém 66075-110, Brazil; ecostag@ufpa.br

**Keywords:** cyanobacteria, oligopeptide, microviridin, biotechnology, ecology

## Abstract

Cyanobacteria are a rich source of secondary metabolites with a vast biotechnological potential. These compounds have intrigued the scientific community due their uniqueness and diversity, which is guaranteed by a rich enzymatic apparatus. The ribosomally synthesized and post-translationally modified peptides (RiPPs) are among the most promising metabolite groups derived from cyanobacteria. They are interested in numerous biological and ecological processes, many of which are entirely unknown. Microviridins are among the most recognized class of ribosomal peptides formed by cyanobacteria. These oligopeptides are potent inhibitors of protease; thus, they can be used for drug development and the control of mosquitoes. They also play a key ecological role in the defense of cyanobacteria against microcrustaceans. The purpose of this review is to systematically identify the key characteristics of microviridins, including its chemical structure and biosynthesis, as well as its biotechnological and ecological significance.

## 1. Introduction

Cyanobacteria are among the first living beings to exist on Earth. The oldest fossil cyanobacteria registries date back 3.8 billion years ago. Their presence was crucial to the creation of an aerobic atmosphere, resulting in the emergence of an enormous species variety [1]. They are defined as prokaryotic oxygen photosynthetic microorganisms and are mainly known for their ability to synthesize structurally diverse and biologically active natural products [2]. In addition, similar to other bacteria, these microorganisms are nucleus-free and have an immense morphological diversity. The various structural shapes encountered in these species are the result of their ability to alter their morphology according to the environment allowing for higher energy accumulation and growth [3,4].

These microorganisms are at mercy of various stress situations found in diverse types of environments, including water-based and land-based. The ability to thrive in these heterogenous environments can be attributed to an enormous secondary metabolite repertory, which has intrigued numerous scientists for its rarity and richness [5,6]. Peptides generated by ribosomal synthesis and produced by large multi-domain enzymes called nonribosomal peptide synthetases (NRPS) are among these metabolites [7,8]. The macrolides present in these photosynthetic species derive from an enzyme complex called polyketide synthase, which is also modular in nature, similar to animal fatty acid synthase. Some molecules are synthesized from the combination of these two metabolic pathways, such as toxin nodularin and microcystin. Products from these two pathways constitute the majority of the secondary metabolites described in cyanobacteria [9].

The ribosomal peptide pathway forms a group very diverse and complex of products, and it is present in all three domains of life. The building blocks used by this pathway are usually limited to 20 proteinogenic amino acids. The enormous structural diversity of these proteinaceous substances can be enriched by post-translational modifications, which are also responsible for the functional diversity contained in this category. Such modifications occur in the side chains and can lead to different forms of macrocyclization [10,11]. The precursor peptide is mainly formed by a leader peptide (LP) and core peptides (CP), which act as recognition and modification sites, respectively. This identification assists post-translational enzymes to focus a biosynthetic effort on a particular precursor peptide. The different types of post-translational modifications (PTMs) are used to differentiate the subfamilies of this group and can enhance the stability of the peptide and its activities [12,13].

Microviridins are among the most promising peptides found in cyanobacteria. These molecules are potent inhibitors of protease found in an enormous variety of cyanobacteria, mainly those of the genus *Microcystis*, *Planktothrix*, *Anabaena*, *Nostoc* and *Nodularia* [14]. An in silico analysis revealed that the occurrence of microviridins in bacteria belonged to other phyla [15,16]. Here, we present a review of the microviridins produced by cyanobacteria and their biotechnological and ecological relevance.

## 2. Microviridin

**Figure 1:**
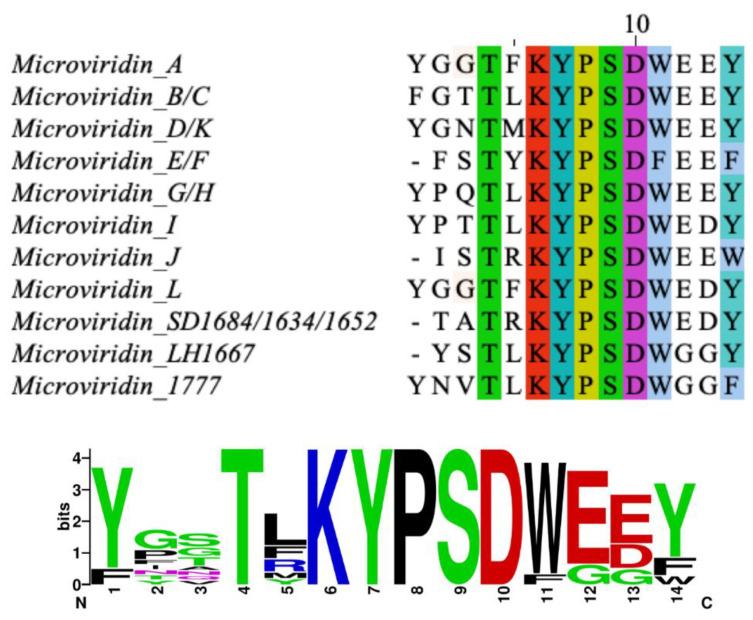
Diversity of microviridin sequences and the conserved KYPSD motif. Multiple alignment was obtained by Clustal Omega (https://www.ebi.ac.uk) and visualized using JalView software (https://www.jalview.org), and the consensus sequence was generated by WebLogo (https://weblogo.berkeley.edu).

Microviridins are one of the most known and largest oligopeptides formed by cyanobacteria. They are ribosomally produced, classified as depsipeptides. Their size can vary from 12 to 20 amino acids, where the N-terminal residue is typically acetylated [17,18,19]. By post-translational modifications, the side chains of some of these amino acids lead to ω-ester and an ω-amide linkage, which result in distinct ring formations. When completely cyclic, microviridins typically exhibit two ester bonds between the Thr-Asp/Glu and Ser-Asp/Glu side chains and an amide bond formed between the Lys side chain at position 9 and Glu or Asp at position 2. The formation of amide and ester bonds are catalyzed by ATP-grasp enzymes. Mono- and bicyclical structures may also be formed, possibly due to the lack of one of the PTM enzymes or further modification of the tricyclic microviridin [14,15,20]. These oligopeptides are capable of inhibiting the hydrolytic activity of several serine protease, including elastase, trypsin, thrombin and chymotrypsin, as well as tyrosinase. Hence, they have cogitated as promising agent in the treatment of several metabolic disorders [21,22]. Their selectivity can be related to their amino acid sequence, especially that occupying the fifth position from the C-terminal. All known microviridins normally share the TxKxPSD motif and possess Asp, Thr, Ser and Lys residues (Figure 1) [20]. 

Microviridins have been identified in different cyanobacterial genera, mostly isolated from freshwater. The screening of environmental samples and isolated strains showed a wide distribution and diversity of this oligopeptide [14]. The majority of reports focused mainly on the strains of *Microcystis* and *Planktothrix*, as these genera are bloom-forming and are usually found in the eutrophic ambient. Over the last few years, more microviridin variants have been discovered in phyla other than cyanobacteria [15,16]. 

## 3. Microviridin Structure

**Figure 2:**
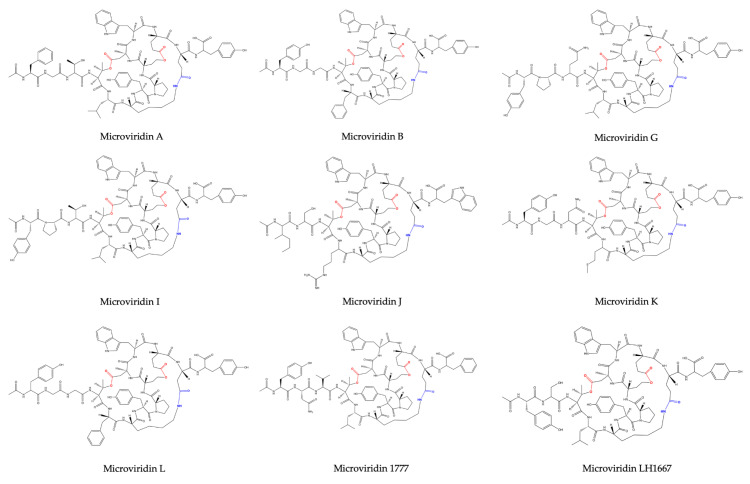

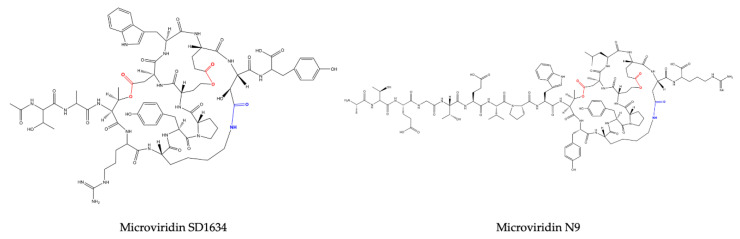
Microviridin structures belonging to group I.

Microviridin was firstly described in the toxic *Microcystis viridis* (NIES-102), which was isolated from a bloom on Kasumigaura Lake, by Ishitsuka et al. (1990) [21]. Its amino acid sequence was defined as Ac-Tyr (I)-Gly (I)-Gly (I)-Thr-Phe-Lys-Tyr (II)-Pro-Ser-Asp-Trp-Glu (I)-Glu (II)-Tyr-OH, where Lys is bound to Glu (II) through its ε-NH with γ-CO of Glu (II). Thr and Ser amino acids are esterified and form ester bonds with the γ and δ carboxylic moieties of Asp and Glu (I), respectively (Figure 2). After the discovery of microviridin A, Okino et al. (1995) [23] identified a further two novel microviridins in the freshwater cyanobacterium *M. aeruginosa* (NIES-298). They were named microviridin B and C, the former exhibiting high similarity to microviridin A. They differ solely by three amino acid residues: Phe, Thr and Leu, which occupy the same position of Tyr (I), Gly (I) and Phe in microviridin A. The microviridin B amino acid composition was defined as Ac-Phe-Gly-Thr-(I)-Thr (II)-Leu-Lys-Tyr-Pro-Ser-Asp-Trp-Glu-(I)-Glu (II)-Tyr-OH. Microviridin C is closely related to microviridin B, exhibiting the same amino acid composition but containing a methoxy group in the γ carboxylic acid of Glu (I) and one additional hydroxyl group correlated to Ser. In this oligopeptide, neither Ser nor Glu are esterified. The slight difference between anti-elastase activity exhibited by both inhibitors was important to demonstrate that the ester bond between Ser and Glu(I) is not included in the reactive site. 

One year later, Shin et al. (1996) [24] revealed the presence of three novel microviridins in *Planktothrix agardhii* (NIES-2014), known as microviridins D, E and F. Microviridin D is a bicyclic peptide, the N-terminal of which is occupied by an acetylated Tyr. Similar to microviridin A, this metabolite also possesses a ester bond formed between the side chains of the Thr and Asp residues. Differing from the former, microviridin D has Asn and Met residues instead of Gly and Phe, respectively. Furthermore, the ester bond between the γ-carboxyl of the Glu and the Ser hydroxyl group is missing in microviridin D, since γ-carboxyl of the Glu existed as a methyl ester. Microviridin E was the first microviridin composed of 13 amino acids described. In microviridin E, three Phe residues replaced two Tyr and one Trp residues of microviridin D. Unlike the other microviridins mentioned above, which have Glu occupying the second position from the C-terminal, this oligopeptide presents the residue of Asp in this position. Microviridin F seems to be a hydrolyzed microviridin E product with the same amino acid sequence. The absence of an ester bond between Thr and Asp is the main difference compared to other microviridins mentioned above. *Nostoc minutum* (NIES-26) was uncovered in 1997 as a source of two novels microviridins (G and H). Microviridin G is structurally related to microviridins A and B, while microviridin H has its structure closely related to microviridin C. These newly identified peptides have the same amino acid compositions. However, microviridin H does not have an ester bond between the Ser and Glu amino acid residues [25]. 

Microviridin I was firstly identified in the nontoxic *P. agardhii* strains 2 and 18. This oligopeptide exhibits high similarity to microviridins A, B and G. They share the Lys-Tyr (2)-Pro (2)-Ser-Asp (1)-Trp-Glu amino acid sequence, as can be seen in Figure 1 [26]. Microviridin J was firstly described in *M. aeruginosa* strain UWOCC MRC, being composed of 13 amino acids organized in three rings and two linear side chains. Unlike the previous microviridins, this peptide has arginine residues between Thr and Lys, which confer a special arrangement with the hydrophobic regions formed between the side chain of this residue and other amino acid residues. This novel structure conferred by the Arg residue occupying the fifth position provides ring stabilization and may be associated with a strong inhibition of trypsin, which has been identified solely in this microviridin [27]. The N-acetyl group of microviridin J also contributes to a marginal increase in the inhibition of trypsin by hydrogen bond formation [28]. The greatest amount of this toxin was obtained by utilizing MeOH at a concentration between 40–80%. The lowest yield was achieved by utilizing absolute methanol [27].

Reshef and Carmeli (2006) [29] isolated, for the first time, three microviridins with the nonproteinogenic amino acid β-hydroxyaspartic acid (Has) bound to lysine through an amide bond. These oligopeptides received the names of microviridin SD1684, SD1634 and SD1652 and were isolated from the extract of *M. aeruginosa* (IL-215). All these microviridins exhibit the same amino acid compositions. However, they differ regarding the number of ester bonds. SD1684 has no ester bonds (solely the amide bond), while SD1634 possesses the two-ester bonds and SD1552 contains only one ester bond, Ser-Glu. 

Vegman and Carmeli (2014) [30] isolated from the extract of a yellow-brown bloom material composed of *Microcystis* spp. (TAU IL-376) the microviridin LH1667, whose amino acid sequence was defined as Ac-Tyr (I)-Ser(I)-Thr-Leu-Lys-Tyr (II)-Pro-Ser (II)-Asp-Trp-Glu(I)-Glu (II)-Tyr (III), with a Lys side chain amine and Glu (II) side chain carboxylic acid connected via a lactam, Ser (II) side chain hydroxyl and Glu (I) side chain carboxylic acid connected via a lactone and a side chain of Thr forming a lactone ring with a side chain carboxylic acid of Asp [30].

The increased number of genome sequences belonging to cyanobacteria opened the doors to a deeper knowledge about microviridins, allowing the discovery and engineering of new variants. The structure of microviridin K was determined by Philmus et al (2008) [15] in *P. agardhii* CYA126/8. Its amino acid composition is similar to microviridin D. However, the residue of Glu12 is not methylated. This oligopeptide thus contains two rings of lactone. Microviridin L, detected in cyanobacterium *M. Aeruginosa* (NIES843), was one of the first cyanobacterial oligopeptides to be characterized with the assistance of genomic data. The gene cluster of this metabolite was inserted into a fosmid and subsequently expressed in *Escherichia coli* [31]. 

Microviridins N3−N9 were identified in the model strain *N. punctiforme* PCC73102 via a genomic approach. These unusual microviridins contain between 15 and 20 amino acid residues and are not acetylated. The name was given to highlight the difference between the number of N-terminal amino acids, which can range from three to nine [19].

Two new microviridins have recently been discovered in strain *M. aeruginosa* EAWAG 127A: microviridin 1777 and microviridin O [32]. The former is the most potent chymotrypsin inhibitor of the microviridin class, while the latter was not detected in the extract, although the precursor peptide gene was contained in the genome (EZJ55 03525). An antiSMASH analysis allowed the identification of its gene cluster. This oligopeptide exhibits high similarly with microviridins A, B, G and J. They share the Lys-Tyr (2)-Pro (2)-Ser-Asp (1)-Trp-Glu amino acid sequence. Its peptide sequence is AC-Tyr-Asn-Val-Thr-Leu-Lys-Tyr-Pro-Ser-Asp-Trp-Glu-Glu-Phe.

**Figure 3:**
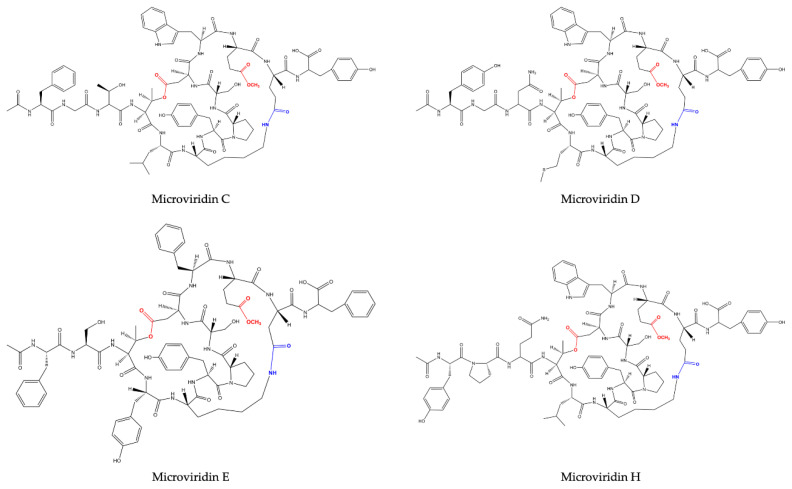
Microviridin structures belonging to group II.

**Figure 4:**
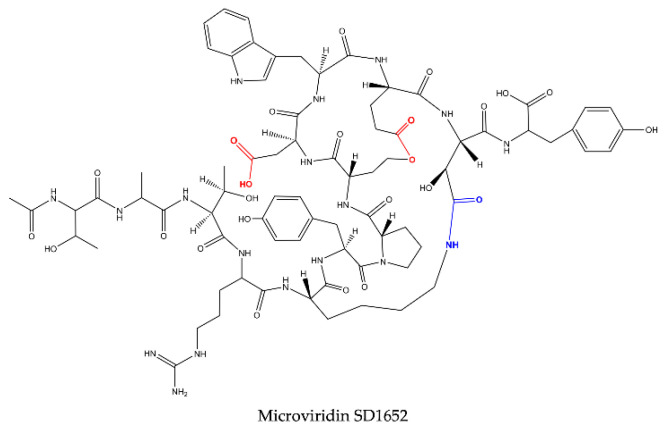
Microviridin structure belonging to group III.

**Figure 5:**
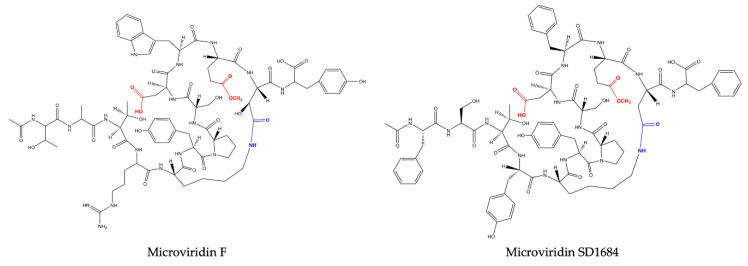
Microviridin structure belonging to group IV.

Based on the number and structure of the ester bonds, microviridins can be classified into four classes. The amide bond is conserved in all of them. Group I consists of microviridins with two ester bonds. The second and third groups have only one ester bond between Thr1-Asp7 and Ser6-Glu9, respectively. In the fourth, microviridins are present with only the amide bond conserved (Figure 2, Figure 3, Figure 4 and Figure 5).

## 4. Microviridin Biosynthesis

Owing to their atypical conformation, microviridins have been mistakenly labeled as nonribosomal peptides. This concept has been discarded, because numerous studies have failed in the quest for biosynthetic gene clusters with mechanisms linked to NRPS genes and being similar to ribosomally biosynthesized peptides, such as cyanobactins (patellamides, tencyclamides and patellins) and trichamide [15,33]. In addition, NRPS products usually have nonproteinogenic amino acids in their structure and can be paired with hydroxy acids. Furthermore, their amino acids can also be in a D-configuration. These characteristics are not usually present in the family of microviridins [15,33]. Microviridins have recently been identified as ω-ester-containing peptides, along with plesiocins and thuringinins of the ribosomally synthesized and post-translationally modified peptide (RiPP) family [34].

Apart from the fact that microviridins have been isolated and characterized since the 1990s, their biosynthesis started to be elucidated by two groups independently using separate approaches in 2008 [14,15]. Firstly, Ziemert et al. [14] pursued a NRPS gene cluster related to microviridin production in *Anabaena*; however, they detected a gene with similar sequence to microviridin, known as *mdnA*. In the immediate proximity of *mdnA*, two additional genes were discovered, named *mdnB* and *mdnC* [14]. In comparison, Philmus et al. 2008 [15] detected similar genes from *Planktothrix agardhii*. This filamentous cyanobacterium possesses a homologous *mdnA* sequence, named *mvdE*, and homologous genes of *mdnB* and *C* encoding two ATP-grasp ligases (*mvdB* and *mvdC*). In addition, an acetyl transferase (*mvdB*) and an ATP-binding cassette transporter (*mvdA*) were detected, which their homologous genes were identified in *Microcystis* named *mdnD* and *mdnE*, respectively (Table 1) [15].

**Figure 6:**
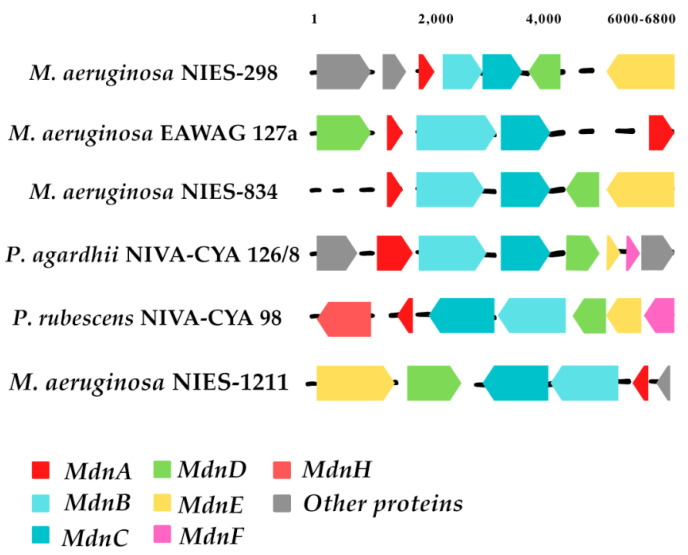
Graphical representation of microviridin biosynthetic clusters. The gene cluster compilation was accomplished through the Gene Graphics application (https://katlabs.cc/genegraphics/app).

These genes have been analyzed by various methods, confirming their roles during the synthesis of microviridins. The heterologous expression of microviridin B *mdnA-C* genes from *Microcystis* in *E. coli* produced a tricyclic microviridin-lacking leader peptide [14]. Concurrently, the in vitro reconstitution of the MvdB-E enzymes from *P. agardhii* also confirmed that these genes were linked to the production of microviridins [15]. These studies were important to demonstrate that the microviridin biosynthetic clusters have different organizations, with or without different genes (Figure 6) [14,15,35].

Through an extensive bioinformatics study of microviridin biosynthetic gene clusters, a number of variations between them have been identified. The majority of these clusters consisted of *mdnA-C* genes, where *mdnB* and -*C* are normally in strict order. However, *mdnD* is only present in a subset of the clusters found. In comparison, *mdnE* is also absent in microviridin gene clusters or replaced by the C39 peptidase, which is followed by the HlyD3 homolog protein, normally linked to the transport of proteases across membranes. Several other gene clusters carry additional proteins, likely linked to the noncommon post-translational modification of the core sequence, such as *mdnF* and *G* [21,33].

**Figure 7:**
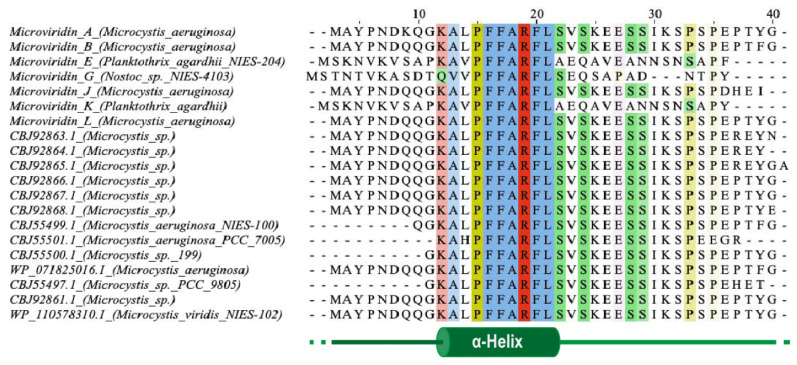
Leader peptide sequences from different microviridins. The PFFARFL motif is highly conversed among them. This sequence and some of its flanking amino acids are structured as an α-helix, responsible for recognition by ATP-grasp ligases. Multiple alignment was obtained by Clustal Omega (https://www.ebi.ac.uk) and visualized using JalView software (https://www.jalview.org).

One of the first steps needed to produce a completely tricyclic N-acetylated microviridin is the production of prepeptide. The microviridin precursor gene (*mdnA*) produces an immature peptide that its leader peptide (LP) has preserved among different variants and possessing a highly conserved PFFARFL motif among the microviridin gene clusters, which has a α-helix structure in a solution (Figure 7) [36]. The core sequence frequently contains Asp, Thr, Ser and Lys residues, as well as the TxKxPSD motif, in which both features are related, to form lactone and lactam rings [20]. When evaluating different cyclized peptides with ω-ester and ω-amide bonds, their core sequences have a high frequency of conserved Thr and Glu residues, which are highly related to lactone ring formations. In addition, when contrasting plesiocin, thuringinins and microviridins, the residues involved in both ester and amide bonds are arranged in a similar order: the nucleophilic residues (Lys, Ser and Thr) always precede the acidic residues (Glu or Asp), indicating their relationship to the directionality of the modification enzymes, as described below [34].

**Figure 8:**
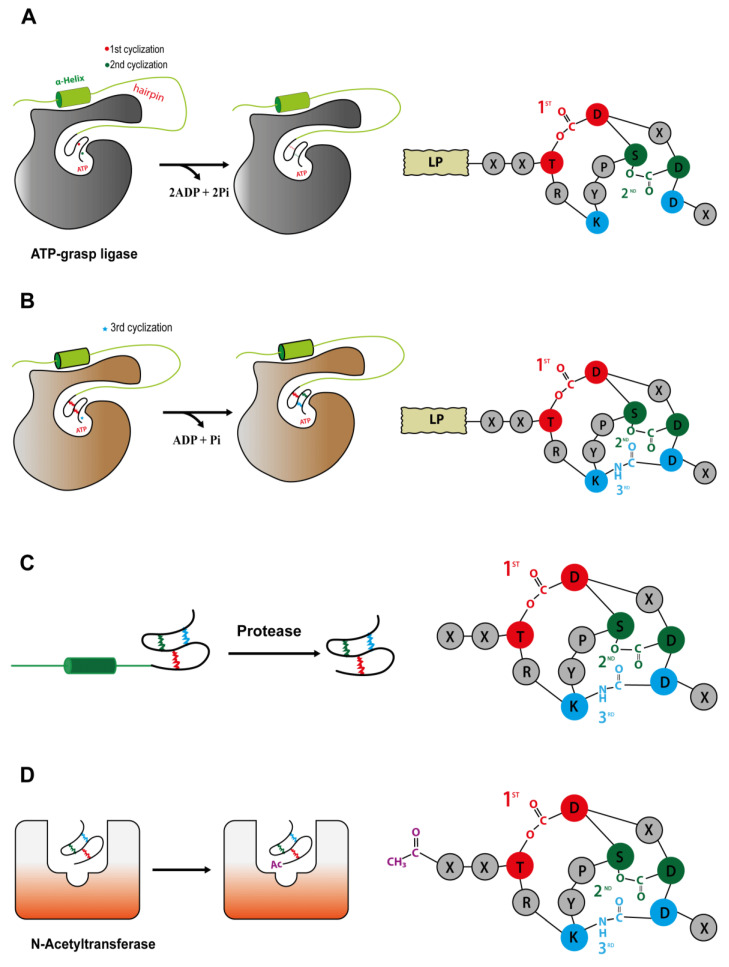
Microviridin biosynthesis. (**A**) Ester bond formations by MvdD/MdnC. (**B**) Amide bond formations by MvdC/MdnB. (**C**) Removal of a peptide leader by a proteolytic enzyme. (**D**) N-acetylation by MvdB/MdnD.

The PFFARFL motif and its α-helix structure is crucial as a recognition motif for the ATP grasp-type ligases (MdnB and -C), as can be visualized in Figure 8A, considering that both enzymes do not modify the core microviridin peptide when the leader peptide is absent, and lactonization and lactamization occur with the PFFARFL motif presence [36,37,38]. The PFFARFL motif is also present in the leader peptide of marinostatin, a double-cyclic peptide with serine protease inhibitor activity, which, by a phylogenetic analysis, suggests that this bicyclic peptide is derived from microviridins [20,37]. Nevertheless, the N-terminal ten-residues sequence of MdnA is not relevant for MdnB and C activity, as this modified prepeptide still containing a PFFARFL motif can also be cyclized and processed. However, a N-His_6_-tagged MdnA with an integral LP fused to three consecutives core peptides was not able to be processed by MdnC from *Anabaena* sp. PCC7120 [36,39].

In addition to this motif, a proline-rich segment is present in the C-terminal region of the leader peptide in a variety of microviridins from *Microcystis* organisms, close to those eukaryotic signal peptides normally associated with cleavage sites. In contrast, microviridin K obtained from *Planktothrix aghardii* CYA128/8 possesses only one proline at the same region [15,35,40]. However, the substitution of these prolines in *Microcystis* did not affect the removal of the peptide leader but resulted in the cessation of microviridin production [37], suggesting the necessity of the β-turn of peptide leaders MdnB and C.

Ahmed et al [20] analyzed several *mdnA* sequences among their biosynthetic clusters and divided this gene into three different classes. Class I precursor peptides contain the LP fused to only one core sequence and are often associated with the presence of *mdnE*, which occurs in a majority of the strains. Class II precursor peptides present a single leader peptide for up to five core peptides in tandem, separated or not by double-glycine cleavage sites, and these clusters normally encode a C39 peptidase membrane protein. Finally, class III is identical in length to class II, but the former has its core sequence only at the C-terminal of the prepeptide [20]. This indicates a number of pathways for the genetic organization of *mdnA*. 

After *mdnA* has been expressed, prepeptides should be submitted for cycling by the sequential catalysis of the enzymes. Thus, in order to understand the mechanisms related to this stage, Philmus et al. [15,37] were the first to define, through biochemical methods, the steps taken by MvdC and D of *P. agardhii* CYA126/8. Both enzymes are carboxylate-amine/thiol ligases that belong to the ATP-grasp superfamily and act by requiring ATP and Mg^2+^ [38,40] to form a carboxylate–phosphate intermediate, which is then susceptible to nucleophilic attacks to form ester, amide or thioester bonds [17,36]. MvdD/MdnC are responsible for the first step in the formation of both ester bonds (Figure 8A) and, subsequently, lactone rings in the linear prepeptide, while MvdC/MdnB are responsible for the formation of lactam rings by amide bonds [15,35].

Both enzymes are homodimers with related assemblies, similar to most proteins of this family, having three subdomains: N-domain, central domain and C-domain. Besides their overall similarities, there are differences comparing their central and C-domains. MdnC/MvdD possess a two-stranded antiparallel β-sheet forming a hairpin structure, followed by a reasonably ordered α-helix that anchors the leader peptide. Meanwhile, this hairpin region is located at the C-domain of MdnB/MvdC, followed by a flexible loop in the α-helix region. The MdnB has a closed conformation, compared to MdnC, because the antiparallel β-sheet hairpin blocks the pocket site where MdnA interacts. Those differences can be related to their specificity and mode of action, as can be seen below. Regarding the ATP-binding pocket, it is structurally conserved, as confirmed by mutagenesis, where substitution of the key amino acids completely abolished the MdnC reaction [36].

Phylogenetic studies and the study of preserved sequences of different classes of prepeptides forming cyclic structures by the action of ATP-grasp ligases (plesiocins, microviridins and thuringinins) suggest that the enzymes coevolved with their respective precursor peptides due to the specificity of the preserved residues present in the core sequence [36]. Consequently, the association between microviridin production and ATP-grasp enzymes indicates that cyanobacteria recycled primary metabolic enzymes for the production of natural products, such as ribosomal peptides, as most ATP-grasp ligases are engaged to primary metabolism [15,17,35,36]. In addition, MdnC is well-conserved among *Microcystis* species, suggesting its derivation from a common ancestor, as well as its dependence on the core motif KYPSD and threonine and aspartate conservation sites of microviridins, as seen by the mutagenesis and phylogenetic analysis [14,15].

As described by Li et al. (2016) [36], the reaction of the bond formation by MdnC (Protein Data Bank (PDB) code 5IG9) is driven by the interaction with the leader peptide. Thus, the PFFARFL motif structured as a α-helix and its flanking amino acids interact with the MdnC hairpin, inducing its movement towards the linear prepeptide bound to the enzyme, then acting as an allosteric region. Considering the ATP-grasp ligase from *Microcystis aeruginosa*, these interactions occur between the amino acids Arg17 (MdnA) and Glu191, Asp192 and Asn195 (MdnC) and Ser20 (MdnA) and Val182 (MdnC). However, Glu191 is mostly replaced by an aspartate residue among all microviridin macrocyclases but still bears the negative charge required to recognize the LP [36].

After binding to the peptide leader through the PFFARFL motif, the ester bond formations are strictly required to occur in a specific order in microviridins: MvdD catalyze the lactone ring between Asp44 and Thr38, then Glu46 and Ser43 into the prepeptide, by phosphorylating the carboxyl side chain of Asp and Glu with ATP, thus forming the large then small lactone rings, respectively [37]. These residues participating in the amide bond and ring formation are highly conserved among the cyclic peptides, suggesting their requirement for the correct cyclization and similar catalysis between ATP-grasp ligases from different groups [34].

When a site-directed mutagenesis was applied to produce different variants of MvdE/MdnA, S43A and T38A, it has been noticed that MvdD catalyzes a reaction following a N-terminal-to-C-terminal direction, as the S43A variant is still lactonized, producing a monocyclic microviridin. In addition, the amino acid bearing the hydroxyl group is crucial for the reaction, as it seems that MvdD cannot react when it is moved one position in either the N- or C-terminal direction [15,37,41]. It seems that both ATP-grasp ligases are highly tolerant for nonconserved residues, then being able to catalyze different microviridins. However, they are not flexible to conserved residues that are involved in cyclization [34,35]. 

For a better understanding of the different MdnC/MvdD enzymes, Zhang et al. [39] characterized a homolog of these enzymes from *Anabaena* sp. PCC7120, AMdnC, which belonged to a biosynthetic cluster with a prepeptide of class II, with a LP followed by three consecutives core sequences (AMdnA). The mode of action of AMdnC indicates a distributive catalysis, where the ATP-grasp ligase dissociates from the processed peptide after each monocyclization, until achieving all lactone ring formations. This feature has been also described in other modification proteins from RiPP pathways, such as the NisB, LctM, LabKC and HalM2 enzymes from lanthipeptides processing; microcin B17 synthetases; ATP-grasp enzyme PsnB and N-methylation enzyme OphA of omphalotin. Additionally, AMdnC also demonstrates a preferential N-to-C directionality when catalyzing the reaction but not unstrict. Thus, this homolog of MdnC can process each core peptide independently from AMdnA. Moreover, the calculated K_m_ from AMdnC when catalyzing AMdnA or MdnA is comparable to MdnC values when processing MdnA; however, the *k*_cat_ of the ATP hydrolysis of AMdnC were up to 60 times faster, suggesting a different mechanism for processing a prepeptide with multiple core sequences.

MdnB has a similar structure and mechanism of activation as MdnC, where the PFFARFL motif interacts with the hairpin, resulting in the activation of the enzyme [36]. Then, the bicyclic prepeptide produced by MvdD/MdnC is catalyzed by MndB/MvdC, and the lactam ring is formed through the amide bond formation between the ε-amino group of Lys40 and δ-carboxyl group of Glu47 (Figure 8B). The omega-amide bond is similar to those present in microcin J25 and capistruin; however, the enzymatic mechanism is different, because microviridin K synthesis occurs via an acyl-adenylated intermediate [17].

Both preformed lactone rings are required by MvdC/MdnB, as the linear and monocyclic peptides are not modified. A single mutation in the PFFARFL pattern in the leader peptide prevents the formation of amide bonds, as well as the proper conformation of the β-turn by the proline-rich region at the C-terminal of microviridin from *Microcystis*, suggesting a lower flexibility compared to MndC [39]. In addition, the amino acid sequence of the core peptide can also influence the correct cyclization, even those not well-conserved, requiring the TxKxPSD motif and Lys and Glu residues [41].

MdnC/MvdD is less rigid than MdnB/MvdC, as it can still catalyze both lactone rings besides single and double mutations in the PFFARFL motif and proline-rich region of the leader peptide (in *Microcystis*) but could result in producing different microviridin variants differing at the N-terminal [37]. This versatility is possibly due to the more open conformation of the hairpin structure and to the binding interaction between MdnC and the prepeptide relative to MdnB. As seen in vitro, the binding interaction between LP and MdnC is approximately tenfold higher compared to the LP and MdnB, resulting in a rapid processing of the linear prepeptide compared to the bicyclic modification. It is also believed that this is due to the fact that linear MdnA is less stable, requiring prompt modification [36]. However, this post-translational modification does not seem to be strictly sufficient for further steps, as bicyclic microviridins can still be cleaved and N-acetylated and possessing inhibitory activity against proteases [41]. As seen by bioinformatic analyses, the absence of MdnB is normal and is likely to lead to the formation of marinostatin, a peptide that lacks an amide bond and is closely related to microviridins [20]. Firstly, it was suggested that MdnE, an ABC transporter, could be related to the removal of the leader peptide through peptide cleavage due to the presence of a N-terminal C39 peptidase domain from *Anabaena* PCC7120 [14,37]. However, not all MdnE carry that domain, and the heterologous expression of the microviridin cluster lacking this protein still produces this tricyclic peptide, indicating other roles [14,37]. Comparing the microviridin expression with the presence and absence of MdnE, it was noted that these peptides were not correctly processed at the N-terminal and were incompletely cyclized due to a lack of lactam rings, as was the amount of MdnB observed in the cytoplasmic fraction when the ABC transporter was absent. This pattern then suggests the hypothesis that MdnE is a scaffolding protein, anchoring and stabilizing the microviridin biosynthesis complex on the cytosolic side of the membrane [37]. In addition to its similarity to transporter proteins, its function in exporting microviridin from the cell has not been demonstrated.

Knowledge on the removal of a peptide leader has so far been scarce in the literature. However, the heterologous expression of microviridin suggests that this step can be mediated by a nonspecific proteolytic enzyme (Figure 8C), as *E. coli* expressing only MdnA-C was capable of producing microviridin lacking a LP [14]. Moreover, GluC endoprotease is capable of cleaving peptide bond C-terminals to glutamic acid residues, and during the in vitro production of class II microviridin with three core sequences, this enzyme released all three mono-, bi- and tricyclized microviridins [40]. Finally, another hypothesis related to MdnE was raised. As described above, this enzyme may have a peptidase domain, typically present in class II clusters. This function may be linked to the presence of interspaced regions between the core sequences of MdnA and the release of each individual microviridin [14,20,37,39].

Acetylation is one of the last steps for the development of a fully matured tricyclic microviridin. Microviridin synthesis in vitro has shown that MvdB from *P. aghardii* CYA126/8 does not require the presence of the peptide leader to acetylate the microviridin N-terminal. In addition, MdnD/MvdB can react with mono-, bi- and tricyclic, being more flexible than MdnB and C. Thus, it can be assumed that this step occurs after the peptide leader removal, or this enzyme does not interact with this region (Figure 8D) [38]. In addition, a 12 amino acid-long tricyclic peptide is not N-acetylated by MdnD from *P. aghardii* CYA126/8, but those microviridin with 13/14 amino acids are acetylated, indicating that there a specific size requirement by this enzyme, and it is flexible regarding the core sequence [20], thus suggesting that N-acetylation occurs only after leader peptide removal [15,20,35,38].

## 5. Occurrence

Genome mining has shown that cyanobacteria have the potential to generate much more microviridin than is typically found under normal growth conditions. A study of this type has contributed to the expansion of knowledge on the chemical and genetic diversity of microviridins. They have been detected in various cyanobacterial genera and species, and these microorganisms are notorious producers of different groups of peptides and can be found in many environments, whether in fresh or salt water (Table 2). Due to this great variety, these bacteria have been evaluated for their significant biotechnological potential. The genus *Microcystis* and the species *M. aeruginosa* are the largest producers of microviridins—currently, of the 25 isolated microviridins, 11 belong to the genus *Microcystis*, and eight of these belong to the species *M. aeruginosa* [21,23,27,29,30,31]. Microviridin gene clusters have also been found in genomes of a number of bacteria, such as bacteroidetes and proteobacteria phyla [20].

The genus *Microcystis* was the first to be described in the literature as a cyanobacteria producer of microviridins. This peptide was isolated from the bloom-forming *M. viridis* (NIES-102) on Kasumigaura Lake by Ishitsuka et al. (1990) [21]. This new oligopeptide demonstrated a noncanonical structure and was named microviridin by the name of the viridis species. In addition, other microviridins from the cyanobacteria of the genus *Microcystis* have been identified as microviridin B, C, L, SD1684, SD1634, SD1652, LH1667, 1777, O and M. Each of these microviridins has a considerable inhibition for at least one serine protease, such as elastase or trypsin [21,23,27,29,30,31]. 

An in-situ diversity investigation of the *Microcystis* communities present in lakes located around and in the city of Berlin, Germany demonstrated that 20% of 165 colonies analyzed were capable of producing microviridin. These cyanobacteria were present in almost all investigated areas. The majority of the microviridins producers also synthesized microcystins and cyanopeptolins. The coproduction of microviridin-aeruginosins and -microginins was rarely reported among the strains, being present in only 4% and 2%, respectively. The metabolomic profile of the peptides can be utilized to distinguish *Microcystis* strains with elevated morphological similarity whose visualization in the light microscope is not sufficient to differentiate them [42]. 

Martins and collaborators [43] isolated strains of cyanobacteria *M. aeruginosa* from a large range of lakes, rivers and reservoirs in Portugal. These strains were examined for the presence of secondary metabolites, such as aeruginosins, microviridins and microcystins. In this analysis, 47 strains from different sites were isolated among the identified peptides; microcystin was the most recurrent, appearing in 26 strains, and microviridins were contained in only three. The results of the analysis of the coproduction showed that the strains that produced microviridins did not produce microcystins. In another study, Walker et al [44] isolated the microviridin-producing strains of the *Planktothrix* genus from Maxsee in Germany incapable of producing microcystins.

In a study accomplished by Andreote [45], the purpose of which was to obtain information on the cyanobacterial community present in the phyllosphere of native plants from the Atlantic Forest, identified 40 cyanobacterial strains belonging to the genera *Nostocaceae*, *Desmontosc* and *Chroococcidiopsis* as microviridin producers obtained from *Merostachys neesii* (bamboo), *Euterpe edulis* (palmeira jacura), *Guapira opposita* and *Garcinia gardneriana*. 

Andreote [45] was the pioneer in the identification of these peptides in the *Desmontosc* and *Chroococcidiopsis* genera. To identify the presence of this peptide in the strains, PCR amplifications of the *mdnA*, *mdnB* and *mdnC* genes were performed, which were related to the biosynthetic pathways of the microviridins. The strains *Nostocaceae* sp. CENA358 and CENA376, *Desmonostoc* sp. CENA365 and *Chroococcidiopsis* sp. CENA353 demonstrated the presence of these genes. Other strains lacked at least one of these genes, which did not rule out the synthesis of this peptide by these microorganisms due to the primers utilized that were constructed for strains of *Microcystis*, causing low-amplification performances, which implies that they might have more strains producing microviridins or possessing a biosynthetic cluster [45].

Eleven cyanopeptides from four different groups were reported from samples of cyanobacterial bloom in the Salto Grande reservoir, located in the State of São Paulo, Brazil, including the microviridin variant 1706. Cyanopeptides such as aeroguniosins, microcystins and cyanopeptolin were also detected. The morphological research showed that the bulk of the population of cyanobacteria belonged to the genus *Microcystis* [46].

Variants of microviridins were characterized in two cyanobacteria isolated from Brazilian reservoirs. *R. fernandoii* strain 28 was obtained from the Furnas Reservoir, which is situated in the southeastern region of Brazil and is described as an oligo-to-mesotrophic aquatic environment that receives organic matter contributions from domestic, farming and agriculture wastewaters. The *R. fernandoii* 86 strain was identified in an urban eutrophic reservoir located in the city of Belo Horizonte, Brazil, which suffers a great impact from domestic pollution, industrial sewage. A total of twelve peptides were found in the two strains. In the *R. fernadoii* 28 strain, a microviridin MV-1709 was found, and, in the strain *R. fernadoii* 86, two microviridins were reported, MV-1707 and MV-1739. Along with microviridins, peptides such as microcystins, cyanopeptolin and an unidentified peptide were also detected [47].

## 6. Microviridin Ecology

**Figure 9:**
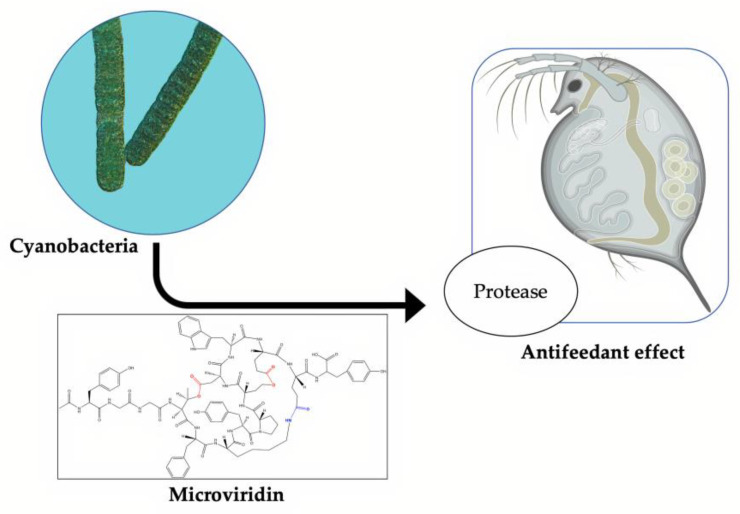
Ecological role of microviridins as antifeedant against the microcrustacean *Daphnia.*

Microviridins play a significant ecological function as antifeeding agents against cyanobacterial natural predators. This activity is correlated with their ability to inhibit proteolytic enzymes (Figure 9). The first study to explain this mechanism was performed by Rohrlack et al. (2014) [48]. A previous work, however, had already indicated microviridins as an agent capable of causing the interrupting the feeding of *Daphnia* microcrustacean via enzymatic inhibition. This ability can partly explain the dominance of these microorganisms in some habitats, including those with a high population density of *Daphnia* [49]. In a similar way, protease inhibitors are produced by terrestrial plants to protect against herbivores. Metatranscriptomic analyses of the Kranji Eutrophic Reservoir, located in Singapore, revealed important information on the functional dynamics between different bacterial phyla, including cyanobacteria, which were dominant microorganisms, especially those belonging to the *Microcystis* genus. The microviridin transcripts were found in high quantities, along with those involved in the buoyancy and photosynthetic operation. The highest peak of the gene expression related to microviridin biosynthesis was observed when the population of *Daphnia* moved from the mesopelagic zone to the epipelagic zone, corroborating its antipredator activity [49].

Kaebernick et al. (2001) [50] compared the feeding inhibition of *Daphnia galeata* and *D. pulicaria* by a microcystin-producing *Microcystis* (MRD) and a microcystin-deficient *Microcystis* (MRC), and it has been realized that this hepatotoxin is not associated with the ingestion rate reduction in both planktonic grazers. However, this metabolite was responsible for causing both species to decrease their survival rates. Before the death provoked by this hepatotoxin, these microcrustaceans remained immobile in the bottom of the vial and shifted only when the surrounding area suffered disturbances. In addition, the filter legs and antenna were momentarily paused, and the midgut was disrupted.

The same authors [50] described further effects of *Microcystis* strain UWOCC MRC ingestion by *D. galeata* and *D. pulicaria*. These microcrustaceans had a dysfunction in the peritrophic membrane. This membrane acts as a barrier formed by the chitin–protein complex created by the midgut cells. The consumption of *Microcystis* made this organ more enervated, as a result of which, food transport was impaired, resulting in particle aggregation in this region and in the digestive diverticula. The ingestion of these cells also disturbed the molting process. The old integument was not entirely separate from the *Daphnia* body, attached to the legs and filter antennas, and strongly hindered the ability of these species to swim and feed themselves. Individuals subjected to these conditions were more likely to die of malnutrition within two days. It was also confirmed that the freshly developed integument remained soft both in the presence of the old integument and after its mechanical removal. Under field conditions, these affected species would become easy prey to predators, since they would not be able to flee to any shelter. 

The typical chitin–protein complex occurs in both structures (peritrophic membrane and integument), indicating that the reported effects were probably caused by the same bioactive compound in which the microviridin variant was cogitated. By its ability to inhibit the serine protease, this oligopeptide could be preventing the tyrosine conversion into dihydroxyphenylalanine (DOPA) and its subsequent transformation into dopamine by the enzyme DOPA decarboxylase [51]. Dopamine is involved in the cross-linkage of orthoquinones, which results in the cuticle sclerotization [52]. A complementary process was proposed by Rohrlack et al. (2003) [48]. According to this mechanism, *Daphnia*’s death was associated with incomplete protein digestion, which resulted in an important amino acid deficiency for tegument development and other structures. Ingestion of the strain of *Microcystis* UWOCC CBS, a producer of microviridin J, causes the same activity in the molting process of *D. pulicaria*. However, several additional findings have been made. Particles derived from food suspensions were found on the entire surface of the *Daphnia* body. This dysfunction was possibly due to the secretion of body fluids. Deformation on the freshly generated tegument has become more intense as the effort to eradicate it by these animals has increased. The same phenomenon was visualized when only the purified microviridin J was added.

Czarnecki et al. (2006) [53] detected eight microviridins distributed in three *Microcystis* strains (HUB08B03, HUB11G02 and HUB19B05) with ability to inhibit trypsin-like activity in the planktonic crustacean *Daphnia*. In addition to microviridins, other classes of protease inhibitors, such as some cyanopeptolins, were found in the extract obtained from these cyanobacteria. This ability of unique cyanobacterium or different cyanobacteria from the same genus can generate a variety of combinations of different oligopeptides with distinct proteolytic targets and inhibitory activity. This feature acts as an evolutionary barrier, preventing the adaptation process among zooplankton population.

Microviridin toxicity was also accessed in the fairy shrimp *Thamnocephalus platyurus*, which belonged to order Anostraca. In the course of searching for natural products with cytotoxicity property, Sieber et al. (2019) [32] detected in the extract of *M. aeruginosa* strain EAWAG 127 deleterious activity against this microcrustacean (LD_50_ = 0.43 mg.mL^−1^). A metabologenomic approach revealed the presence of two novel microviridins: microviridin 1777 and microviridin O. The former showed a LD_50_ value of 95 µM for *Thamnocephalus platyurus*. This activity was ascribed to the strong capacity of this peptide in inhibiting elastase and chymotrypsin activity with an IC_50_ of 160 nM and 100 nM, respectively.

In addition to Cyanobacteria having a low susceptibility to a zooplankton attack, these microorganisms are also the target of various pathogenic bacteria and fungi that play an important role in controlling their growth [54,55]. True zoosporic fungi, commonly known as chytrids, are among the most pathogenic organisms capable of causing a significant number of deaths in the cyanobacterial community [56]. The success of this pathogen in infecting these photosynthetic microorganisms can be attributed to the development of chemotactic zoospores and the presence of rhizoids, which are capable of locating the target and used to extract the nutritional contents, respectively. Oligopeptides produced by cyanobacteria with an inhibitory activity against a predator’s key enzymes is a great defense mechanism. The comparison between the cyanobacterial strain *P. agardhii CYA126/8* with its mutants, each one with a type of disability in producing microcystins, anabaenopeptins or/and microviridins, was conclusive to defining the protective role of these metabolites. The wild strain when incubated with the chytrid strain was unaffected, while all mutant strains were infected, including those non-microviridin-producing strains [57].

Chytrides are a rich source of protease used as a mechanism to digest their hosts. Microviridins and anabaenopeptins can target these enzymes, reducing the virulence of these fungi. The vast variety of microviridins, as well as other oligopeptides, is a major obstacle in the process of the adaptation of these parasites. On the basis of the literature, the protection mechanism referred to above appears to be constitutive, since these substances typically form an oversaturated or saturated solution in the cytoplasm [57]. Microviridin was also found in bacteria belonging to the microbiome of the plant *Populus*. Unlike the lanthipeptides that are widely distributed among the member of this community, microviridins were restricted only to the genus *Chryseobacterium*, being present in 16 out of the 18 sequenced bacteria. Its role in this microbiome is not clear. A gene cluster for microviridin in this genus showed from one to four precursor peptides belonging to class I [20]. Different from cyanobacterial microviridin, the core peptide was composed of 18 amino acid residues. Only half of the microviridin clusters analyzed had a N-acetyltransferase gene. A resistant gene presence in the majority of the microviridin clusters suggested that this oligopeptide could have antibacterial properties, conferring a protection to plants against pathogenic microorganisms [58].

Other features given to microviridins are related to their allelochemical properties. Cyanobacteria produce a variety of proteases that are essential to different processes, including nutrient absorption, protein activation, unfolded or aggregated protein removal, photoacclimation and stress response [59]. Ghosh et al. (2008) [60] demonstrated that a cyanobacterial oligopeptide with the partial structure of a microviridin affected the proteolysis in *M. aeruginosa* PCC 7806, strongly inhibiting its capacity to degrade N-alpha-benzoyl-DL-Arg-p-nitroanilide (BApNA). The authors’ hypothesis was that microviridin-producing *Microcystis* colonies could form an aggregate that could eventually develop as a bloom and suppress the growth of competing organisms by targeting critical functions that rely on protease activity. Another possibility is that microviridins will have a significant role to play in stress conditions by self-regulating the protease activity among cyanobacterial cells and thus enhancing their survival rates [60]. 

Some microviridins are not easily detected in environmental samples, since they may be rapidly degraded by other bacteria. *M. viridis* has its microviridin A content totally consumed when transferred to a nonaxenic medium [21]. The aquatic bacterium *Sphingomonas* sp. B-9, firstly isolated for its microcystin-degradation ability, has hydrolytic enzymes capable of degrading different cyclopeptides, including microviridin I. The degradation of this peptide by this bacterium is very slow, lasting around 48 h to reduce 50% of its initial content. This process occurs in two steps. Initially, the residue at the C-terminal region is removed, and, subsequently, the molecule undergoes a linearization step [61].

## 7. Regulation

Environmental factors play an important role in the regulation of the synthesis of oligopeptides, as they can increase the growth rate and, consequently, the production of these metabolites. In certain cases, however, the best conditions for growth did not lead to the most desirable conditions for their production [62]. Microcystin was the key subject of these studies [63]. Stress situations can alter the cell’s physiological state and act as trigger for increasing the construction of these molecules. Nitrogen and phosphorus bioavailability are among the most nutritional factors investigated in cyanobacterial behaviors [64,65]. Both are involved in protein synthesis and in the energy dynamic. Due to anthropic actions, these elements have become more abundant in the aquatic environment [66].

Other parameters, such as temperature, pH and light intensity, have also been investigated and challenge many scientists [67,68]. An assessment of the combined effect of different environmental elements on the development of cyanopeptides can provide a link between field research and laboratory research. Any of these variables can be associated. As the culture reaches the stationary phase, the quantity of nutrients decreases, as well as the light availability among cells, thereby reducing their growth rate. 

One method used by Rohrlack et al. (2007) [67] to individually determine the light impact was to track and maintain a constant nutrient load in the medium. This technique was used to analyze the production of microviridin I by *P. agardhii* strain PT2. The quantity of cell-bound microviridin I expressed in units per biovolume decreased until the eighth day. This behavior reversed when the light availability began to decline. Nitrogen and phosphate reduction also led to a decrease in the production of this microviridin. A similar trend was reported for microcystins and anabaenopeptins. Some authors strongly believe that many of these oligopeptides play the same ecological function. The loss of one can have, as a consequence, the enhanced production of another, unaffecting the cyanobacterial growth [69,70].

The influence of light intensities was also evaluated by Pereira et al. (2012) [47] on the profiles of toxic and nontoxic oligopeptides obtained from two strains of the cyanobacterium: *R. fernandoii* 28 and 86. In the course of the experiment, they employed three different irradiances, which were classified as low (25 µmol.m^−2^.s^−1^), medium (65 µmol.m^−2^.s^−1^) and high (95 µmol.m^−2^.s^−1^). Different from other oligopeptides investigated in this study, such as microcystins and cyanopeptolins, microviridins were not encountered in all growth conditions. Microviridin 1709 production reached the maximum amount when the cells of strain 28 were exposed to a medium light intensity, while microviridin 1707, identified in strain 86, was detected solely at low light conditions.

Ferreira et al. (2006) [71] evaluated the combination of different light intensities, nutritional contents, temperatures and growth phases on the oligopeptide production in distinct strains of *Microcystis* and *Aphanizomenon*, including microviridins. This protease inhibitor was detected solely in the *Microcystis* strain RST9501. In the absence of nitrate and phosphate, this peptide was produced in higher quantities. In the majority of the cases, the intracellular fraction was responsible for over 80% of the total microviridin pool. At the same nutritional conditions, an atypical behavior was found when the cells were cultivated at 20 °C. In this condition, the intracellular microviridin concentration diminished to 60%.

The cell-to-cell communication is also a factor to be considered in peptide production. When this mechanism is dependent on cell density, it is called quorum sensing. Nealson and Hastings (1979) [72] were pioneers in studying this phenomenon in the Gammaproteobacterium *Vibrio fischeri*. These two scientists were capable of demonstrating that the enzyme luciferase, whose role is to transform chemical energy into light energy, was expressed only at a high cell density, having its production controlled by autoinducer signaling molecules [72]. The most known autoinducers described are the acylated homoserine lactones [73]. The aquatic environment has a natural tendency to dilute the metabolites released by microorganisms. For this reason, some authors believe that oligopeptide production is regulated by quorum sensing [74]. There is little knowledge about this mechanism in cyanobacteria. During a bloom episode, the cyanobacterial population increased significantly, creating a favorable environment for quorum sensing. In this type of situation, the high cell density augments the concentration of signaling molecules in the environment [75].

To evaluate the quorum sensing effect on oligopeptide production, Pereira et al. [74] grew the cyanobacteria in a semicontinuous culture system. Hence, the biomass level and nutritional content were maintained constant. The growth rates of high and low cell density cultures were similar. Microviridin production was detected only in a low cell density culture of *R. fernandoii* (strain R28). In contrast, the microviridins N3-N9 in *N. punctiforme* PCC73102 had their synthesis optimized under high cell density conditions [19]. 

The cyanobacterial lifestyle may also have an effect on its oligopeptide content. Some cyanobacteria are typically located on the water and sediment surface. There are also those with a biphasic lifestyle, where they migrate to the top during the summer and to the bottom during the winter [76]. A comparative genomics of the genus *Planktothrix* with different lifestyles performed by Pancrace et al. (2016) [77] demonstrated that all planktonic strains investigated harbored the microviridin gene cluster. In contrast, in the benthic *Planktothrix*, this gene cluster was absent, with the exception of *Planktothrix* sp. PCC 11201, which is phylogenetically closer to free-living *Planktothrix*.

## 8. Application of Microviridins

**Figure 10:**
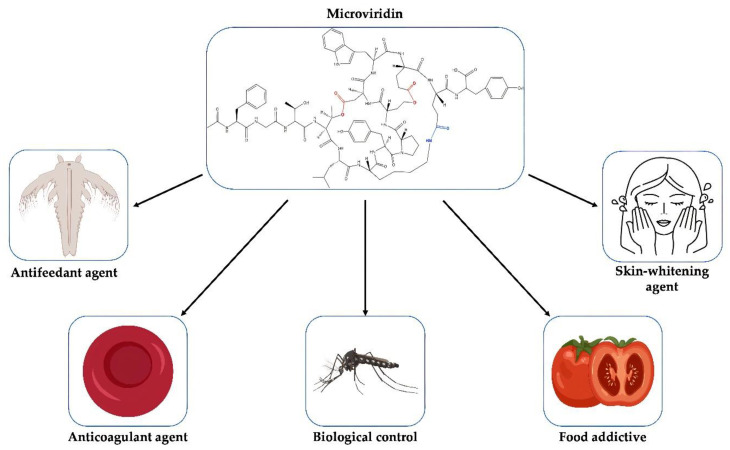
Potential applications of microviridins.

Proteases play an important role in the regulation of biological processes in all living organisms by controlling the maintenance, recovery, development and modification of tissues, which may be beneficial or harmful. They may modulate protein–protein interactions that create bioactive molecules involved in DNA replication and transcription [78]. In plants, these enzymes lead to the maturation and degradation of a series of unique proteins relevant to the environmental condition and the stage of growth [79]. Thus, molecules with inhibitory activity against these enzymes have an immense biotechnological potential with a multitude of applications (Figure 10).

Tyrosinase is a multifunctional and metalloenzyme widely distributed among plants, microorganisms and animals, where it plays a key role in the development of melanin [80]. The excessive synthesis of this photoprotective pigment may lead to a condition known as hyperpigmentation, which may lead to an esthetic problem where one part of the skin is more pigmented than the other [81]. In addition, this disorder has been linked to many diseases, such as skin cancer and Parkinson’s disease [82]. Molecules with the capacity to interfere with the catalytic activity of tyrosinase have been extensively studied as a skin-whitening agent. They can also be used as food additives, reducing the browning process of mushrooms and fruits caused by tyrosinase [83]. Some of these commercially available compounds exhibit low stability and safety [84]. Among the microviridins reported in the literature, microviridin A was demonstrated to be tyrosinase inhibitor. However, its action mechanism is not clear, and information regarding its toxicity to human cells has never been accessed (Table 3) [21]

The mechanism of coagulation is regulated by proteases. Serine protease inhibitors therefore function as essential regulators in this pathway, such as proteins in the serpin superfamily. Malfunctioning of one of these elements can lead to coagulation disorders. Excessive blood clotting can lead to a disorder known as thrombosis, where the blood flow is blocked by thrombus [85]. Thrombin is one the major target of anticoagulant drugs, since it acts in the conversion of soluble fibrinogen into insoluble filamentous of fibrins, which, together with platelets, are responsible for a hemostatic plug formation, impeding the bleeding. The thrombin inhibition by microviridin B is superior to the positive control Leupeptin, possessing an EC_50_ (half maximal effective concentration) value equal to 4.58 µM. This value was inferior than that encountered for Micropeptin K139, a serine protease also detected in cyanobacteria. In contrast, microviridins D-F do not affect thrombin activity, most likely due to the absence of an indole motif, which is encountered in microviridin B, suggesting its role as a recognition motif for thrombin [86].

Human neutrophil elastase is a proteolytic enzyme that belongs to the serum protein family of chymotrypsin-like. This highly active enzyme has revealed a wide substrate specificity and is one of the few proteases capable of degrading the extracellular matrix protein elastin, resulting in the enzyme’s name [87]. The elastase overactivity is involved in tissue destruction and inflammation characteristic of various diseases, such as chronic obstructive hereditary emphysema, pulmonary disease, cystic fibrosis, adult respiratory distress syndrome and ischemic-reperfusion injury [88]. Pharmaceuticals already use elastase inhibitors for the treatment of diseases related to this enzyme, such as the drug Sivelastat, which has been cogitated in the treatment of COVID-19 [89].

A study by Masahiro Murakami et al. (1997) [25] evaluated the elastase inhibitory effect of some microviridins, synthesized by the cyanobacterium *Nostoc insulare* (NIES-26). The results of the analysis showed two new peptides, microviridin G and microviridin H. In the experiment, both IC_50_ were compared with the values of other microviridins already described in the literature. Microviridin A showed no inhibitory effect on elastase; microviridins D and F had the weakest values for inhibiting this enzyme. Microviridins G and H had the best results, followed by microviridins B and C, respectively. After the work of Murakami et al. [25], three new microviridins were described as elastase inhibitors: I, LH1667 and 1777 (Table 3) [26,30,32].

Proteases are also essential for the growth of insects, such as in the larval and adult stages, where they are present in the intestine and play an important role in digestion [90]. For example, silkworms, near the final stage of their metamorphosis, produce cocoonase, a serine protease capable of hydrolyzing silk protein, which enables the adult moth to emerge [91]. During the embryony phase, proteases digest egg-specific proteins, such as vitellin, for the amino acid release, which are utilized as a nitrogen source [92]. Serine protease can also confer protection against predation. The South American Saturniid caterpillars belonging to the genus *Lonomia* harbor in their hemolymph a toxic protease that some mammals who come in contact with can have, as a consequence, bleeding disorders [93]. Nowadays, various studies have focused on the search for a protease inhibitor whose target are proteases produced by insects involved in disease transmission, such as the *Aedes aegypti*, one of the largest vectors of arboviruses, being responsible for the propagation of the dengue virus, yellow fever, zika virus and chikungunya fever [94].

Microviridins have already shown to be good inhibitors of enzymes present in microcrustaceans intestines, such as those of the genus *Daphnia* and *Thamnocephalus* [32,50]. For instance, in the presence of microviridins B, C, I, J, L and SD1652, the enzyme trypsin has its activity negatively affected [23,26,27,29,31] (Table 3). They act by inhibiting enzymes that are closely related to the diet of these crustaceans. In insects, many of these serine proteases are located in the intestines as well [95], sharing similar functions. Two of these serine proteases are trypsin and chymotrypsin, well-known targets of some microviridins (Table 3).

Plants have served as a great heterology expression system for bioactive peptides. Hilder et al. (1987) [96] were the first to use these organisms to express serine protease inhibitors with the potential to kill predatory larvae insects. There is a considerable number of works employing plants as hosts of ribosomally synthesized and post-translationally modified peptides [97,98,99]. Plant-based microviridins have promises for future applications, since they can replace the use of pesticides to help control insect pests with low costs and low environmental impacts. Furthermore, they can be easily purified with a high yield, retaining full activity.

One of the bottlenecks for microviridin production and the evaluation in cyanobacteria is a low yield of this peptide. Several extraction approaches in different genus of this phylum demonstrated that these organisms are not well-suited for industrial applications when considering both the time and volume of the cultivations. A heterologous expression in *E. coli* demonstrated to be an efficient method for microviridin biosynthesis resulted in a yield of 60 °C 70 mg of microviridin L per 100 g of dried cells after five hours of cultivation. In comparison, about 0.87 mg.g^−1^ of microviridin A was obtained from *Microcystis viridis* (NIES-102) by a cultivation period of 10 °C for 14 days. In filamentous cyanobacteria, this yield was even lower, with a production of 9.1 mg.g^−1^ of microviridin E after the same period of incubation of *Planktothrix* in 400 L [21,25,31]. 

However, the problems related to microorganism cultivation, heterologous expression and laborious purification can still be tackled, and techniques have been developed to overcome these barriers in order to explore the diversity of the microviridins. As a consequence, the development of microviridins obtained from environmental DNA can be accomplished by the synthesis of the solid-phase peptide of the core peptide coupling with MdnC and -B enzymes fused to the leader peptide in the N-terminal. This chemoenzymatic approach allows an in vitro production of a fully processed microviridin, demonstrating the efficiency during production of different variants of this peptide [34]. Another approach for microviridin production in vitro is to provide the LP in trans for both MdnB and -C, also achieving a tricyclic microviridin J [36].

Another important feature for the biotechnological application of microviridin is its binding affinity to serine proteases. Microviridin J demonstrated a *K*_D_ value of 0.68 µM from its interaction with trypsin. This mode of inhibition is similar to a cyclic depsipeptide A90720A produced by a nonribosomal peptide synthetase [34,37]. However, the NRPS pathways are not well-susceptible to genetic engineering compared to RiPPs. Thus, in addition to the possibility of genetic modifications, the microviridin biosynthetic cluster is smaller than NRPS, facilitating a heterologous expression [37].

**Figure 11:**
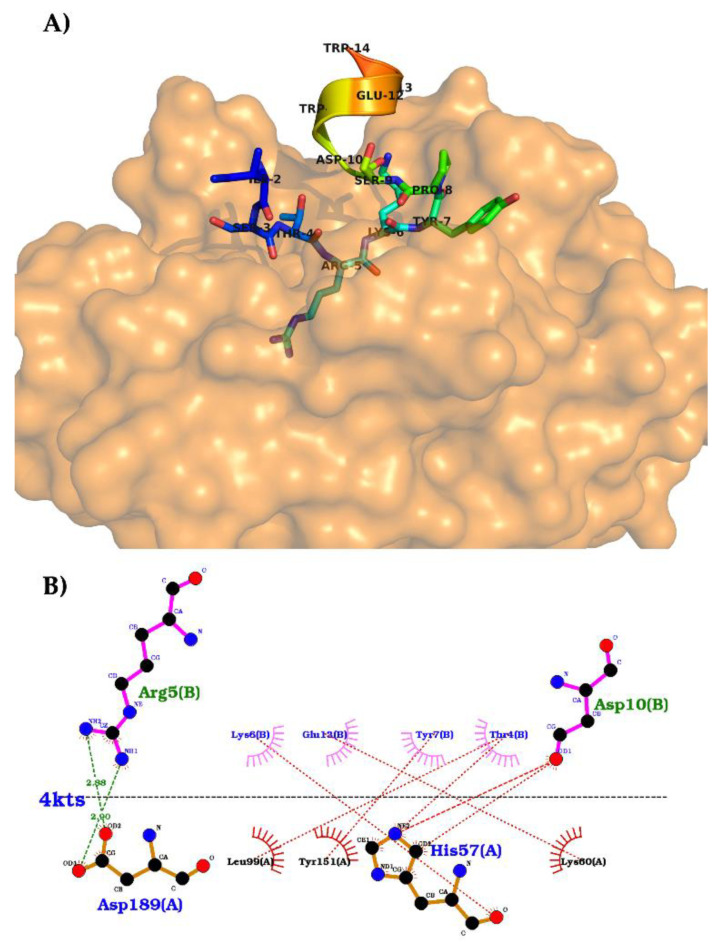
Interaction between microviridin J and trypsin at pH 8.5 (Protein Data Bank (PDB) code: 4KTS). (**A**) 3D representation of the interaction. (**B**) 2D view of the major interaction between microviridin J and trypsin. Hydrogen bonds are in green, while the hydrophobic interactions are in red.

The crystallography structure of trypsin bound to N-acetylated tricyclic microviridin J (PDB codes: 4KTU and 4KTS; pH 6.5 and 8.5, respectively) has been determined to better understand the relationship between the microviridin structure and its enzymatic target (Figure 11). It was therefore possible to observe that the N-terminal of microviridin J was flexible and bound to the hydrophobic surface of trypsin [100]. As far as the catalytic domain is concerned, both crystallized structures showed the interface as a substrate-like trypsin-binding motif. The threonine residue at position 4 of microviridin J interacts with Leu99 at the S2 pocket through its methyl group. At the S1 pocket, Asp189 coordinates the side chain of arginine at position 5, which is located between the residues making ester and amide bonds. A van der Waals contact by the aliphatic region of Lys6 of microviridin is made with the disulfide bond between Cys43 and Cys58 of the S1’ subsite. Finally, the C-terminal of microviridin J, Ser9-Trp14, stabilizes a helical structure of trypsin by the intramolecular covalent linkages of this inhibitor. The interaction between these two structures showed a *K*_D_ value of 0.68 µM, which is similar to the NRPS cyanobacterial inhibitor A90720A [100].

As far as the Ser-His-Asp triad of trypsin is concerned, these three residues are located in the direction of the Arg5 carbonyl of microviridin. However, the peptide bond between Arg5 and Lys6 was not affected, suggesting that the rings and the compact structure of microviridin J neutralize the cleavage of this tricyclic structure [100].

According to the crystallized structures, it could be concluded that position 5 of the microviridins is essential to its inhibitory activity due to the interaction of the trypsin triad. This hypothesis supports the mutagenesis approach to the modification of residue 5 of microviridin L, which modified both the specificity and the inhibitory activity against different proteases. The wild-type microviridin L has its most potent inhibition against subtilisin (IC_50_ = 5.8 µmol.L^−1^); however, the replacement of Phe5 for other amino acids changed this activity. The F5L mutant improved the elastase inhibition; in comparison, F5R increased its inhibition toward trypsin. The F5Y variant shifted its activity against chymotrypsin, and F5M had an IC_50_ = 0.09 µmol.L^−1^ toward subtilisin. In contrast, the exchange of amino acids at positions 7, 9 and 11 did not boost the inhibitory activity at the same scale, nor did any inhibition cease [20,100]. 

The G2A variant coupled with the shift in position 5 of microviridin J not only enhanced the post-translational modification performances but, also, increased the inhibitory function. The positive charged residues of Arg and Lys at position 5 had superior activity towards subtilisin and trypsin, while the latter was the only variant with a low micromolar activity against plasmin [100]. Similar findings were also observed with microviridin B variants L5R and L5K [38]. In addition, the hydrophobic residues of Leu and Val also demonstrated the inhibition of subtilisin and inhibition of elastase inhibitory activity, with minor variations compared to the single mutants at position 5 [100]. As a result, the amino acids at position 5 of the microviridins have shown great potential to be the focus for therapeutic development, with the goal of enhancing and defining the inhibitory action of microviridins by different synthetic chemical techniques.

## 9. Final Considerations

Microviridins are one of the largest oligopeptides present in cyanobacteria. While they were firstly identified in this community of microorganisms, the genomic approach has revealed gene clusters for these metabolites in bacteria belonging to another phyla. Their production is affected by abiotic and biotic factors such as temperature, pH, nutritional content and quorum sensing. This latter is poorly explored in cyanobacteria and can act as a powerful tool in the control of these microorganisms in the environment. Due to their protease inhibitory property, microviridins can be utilized for various purposes, such as an anticoagulant and as whitening agent, as well as in the control of disease vectors.

One of the greatest bottlenecks to the commercial application of microviridins is the low yield and the absence of information about their use in humans and animals. The former issue can be mitigated with the utilization of a heterologous expression system, which has well-described in the literature for this oligopeptide, mainly in the model organism *E. coli.* Others approaches would be the in vitro chemoenzymatic synthesis or the variations in the culture conditions, which could serve as an elicitor, leading to an upregulation of the metabolite. The strong inducible insertion of the promoter may make this process less laborious. In relation to health risks, further studies are required to better develop the inhibitory mechanism of microviridins, as well as their toxicity to humans. However, since they are a ribosomally synthesized and post-translationally modified peptide, they possess a certain plasticity for engineering that can reduce their risks and increase their specificity. The bulk of the cyanobacterial gene cluster remains undiscovered. The ability of these photosynthetic microorganisms to generate biomolecules is greater than was assumed before the genome age. Future studies will disclose new microviridins, as well as knowledge on their biological significance.

## Figures and Tables

**Table 1:** Genes involved in microviridins biosynthesis.

Genes	Product	Role
*mdnA/mvdE*	Microviridin prepeptide	Contains the leader peptide at N-terminal and the core peptide at *C*-terminal
*mdnB/mvdC*	Family of carboxylate-amine/thiol ligases belonging to the ATP-grasp fold superfamily	Lactam rings formation through amide bonds.
*mdnC/mvdD*	Family of carboxylate-amine/thiol ligases belonging to the ATP-grasp fold superfamily	Lactone rings formation through ester bonds
*mdnD/mvdB*	N-acetyltransferase of the GNAT family	Acetylation of microviridin at N-terminal
*mdnE/mvdA*	ATP-binding cassette (ABC) transporter	Stabilization of the biosynthetic enzymes

**Table 2:** Occurrence of microviridins.

Species/Genera	Isolation Source	Trophic State	Sequenced Genome	Microviridins	Mass (Da)	Ref.
*Microcystis. viridis* NIES-102	Aquatic	Eutrophic	+	Microviridin A	1729.7	[21]
*Microcystis. aeruginosa* NIES- 298	Aquatic	Eutrophic	+	Microviridin B	1723.8	[23]
*Microcystis. aeruginosa* NIES-298	Aquatic	Eutrophic	+	Microviridin C	1755.8	[23]
*Planktothrix agardhii* NIES-204	Aquatic	Eutrophic	+	Microviridin D	1802.7	[24]
				Microviridin E	1665.7	
				Microviridin F	1683.7	
*Nostoc minutum* NIES-26	Terrestrial	Mesotrophic	+	Microviridin G	1806	[24]
				Microviridin H	1838	
*Planktothrix agardhii* CYA126/8	Aquatic	N.I.	-	Microviridin K	1769	[15]
*Planktothrix. agardhii* strain 2 & 18	Aquatic	Eutrophic	-	Microviridin I	1764.7	[26]
*Microcystis aeruginosa* UWOCC CBS	Aquatic	N.I.	-	Microviridin J	1684.4	[27]
*Microcystis aeruginosa* NIES-843	Aquatic	Eutrophic	+	Microviridin L	1715	[31]
*Planktothrix* sp.	Aquatic	Eutrophic	-	Microviridin I	1765.8	[43]
				Microviridin 1642	1642.8	
				Microviridin 1663	1663.7	
*Microcystis aeruginosa*	Aquatic	Mesotrophic	-	N.I.	N.I.	[44]
*Microcystis* sp.	Aquatic	N.I.	-	Microviridin LH1667	1666.7	[30]
*Microcystis aeruginosa* PCC 7820	Aquatic	N.I.	-	Microviridin 1706	1707.8	[46]
*Planktothrix rubescens* NIVA-CYA 98	Aquatic	Mesotrophic	+	Microviridin	1971.8	[33]
*Microcystis* sp.	Aquatic	Mesotrophic	-	Microviridin 1667	1668.6	[42]
				Microviridin 1684	1695.8	
				Microviridin 1699	1699.8	
				Microviridin 1777	1778.8	
*Chroococcidiopsis* sp. CENA 353	Leaf Surface	N.I.	-	N.I.	N.I.	[45]
*Desmonostoc* sp. CENA365	Leaf Surface	N.I.	-	N.I.	N.I.	[45]
*Nostocaceae* CENA358	Leaf Surface	N.I.	-	N.I.	N.I.	[45]
*Nostocaceae* CENA376	Leaf Surface	N.I.	-	N.I.	N.I	[45]

N.I.: not informed.

**Table 3:** Inhibitory activities of microviridins.

Microviridin	Inhibitory Activity (IC_50_)							Reference
	Tyrosinase	Elastase	Trypsin	Chymotrypsin	Thrombin	Plasmin	Papain	
A	0.33 mM *	ND	ND	ND	ND	ND	ND	[21]
B	ND	25.5 nM	33.7 µM	1.45 µM	>58.1 µM	>58.1 µM	ND	[23]
C	ND	47.9 nM	18.2 µM	2.8 µM	>57.1 µM	>57.1 µM	ND	[23]
D	ND	388.5 nM	>55.5 µM	665.9 nM	>55.5 µM	>55.5 µM	>55.5 µM	[24]
E	ND	360.1 nM	>60 µM	660.3 nM	>60 µM	>60 µM	>60 µM	[24]
F	ND	3.4 µM	>59.5 µM	>59.5 µM	>59.5 µM	>59.5 µM	>59.5 µM	[24]
G	ND	10.5 nM	>55.4 µM	775.6 nM	>55.4 µM	>55.4 µM	>55.4 µM	[25]
H	ND	16.9 nM	>54.4 µM	1.6 µM	>54.4 µM	>54.4 µM	>54.4 µM	[25]
I	ND	192.7 nM	14.9 µM	12.3 µM	>56.7 µM	ND	ND	[26]
J	ND	>5.9 µM	20–90 nM	1.7 µM	ND	ND	ND	[27]
L	ND	>58 µM	42 µM	58 µM	ND	ND	ND	[31]
1777	ND	160 nM	>10 µM	100 nM	ND	ND	ND	[32]
SD1684	ND	ND	Inactive	Inactive	ND	ND	ND	[29]
SD1634	ND	ND	8.2 µM	15.7 µM	ND	ND	ND	[29]
SD1652	ND	ND	Inactive	Inactive	ND	ND	ND	[29]
LH1667	ND	20 nM	>45.5 µM	2.8 µM	ND	ND	ND	[30]

* IC_50_ not defined. ND, not determined.
